# Improving Bovine Embryo Development and Quality Using Bovine Oviductal Epithelial Cell‐Derived Conditioned Medium (bOEC‐CM)

**DOI:** 10.1002/vms3.70179

**Published:** 2025-03-29

**Authors:** Saeed Kazemi Hosseinabadi, Hassan Nazari, Mehran Arabi, Naser Shams Esfandabadi, Ebrahim Ahmadi, Azita Afzali, Hamidreza Ghanaei

**Affiliations:** ^1^ Department of Animal Physiology Faculty of Basic Sciences Shahrekord University Shahrekord Iran; ^2^ Research Institute of Animal Embryo Technology Shahrekord University Shahrekord Iran; ^3^ Department of Clinical Sciences Faculty of Veterinary Medicine Shahrekord University Shahrekord Iran; ^4^ Clinical Embryologist Shahrekord University of Medical Sciences Shahrekord Iran; ^5^ Faculty of Veterinary Medicine Shahrekord University Shahrekord Iran

**Keywords:** bovine, conditioned medium, embryo, in vitro culture (IVC), oviductal epithelial cells

## Abstract

**Background:**

The embryo co‐culture systems with the monolayer cultured cells are complex, unreproducible, and have a high probability of biological contamination. Therefore, nowadays, using conditioned media is a suitable alternative to these methods.

**Objectives:**

This study aimed to investigate the impact of utilizing bovine oviductal epithelial cell‐derived conditioned medium (bOEC‐CM) on subsequent embryo development and quality.

**Methods:**

Bovine embryos produced in vitro were cultured in a specific medium supplemented with either 5% charcoal‐stripped FBS (Charcoled Strip Serum [CSS]) or 10% bOEC‐CM from either Days 1 or 3 post‐fertilization. Various parameters, such as cleavage rate, blastocyst formation, hatching rate and blastocyst quality, were assessed.

**Results:**

The results indicated that adding 10% CM from Day 1 significantly reduced the cleavage rate compared to using CSS on the same day (*p* < 0.05). Furthermore, the CSS and CM from both Days 1 and 3 increased blastocyst formation rates (*p* < 0.05). Notably, the addition of 10% CM on Day 3 significantly improved the hatching rate compared to the other groups (*p* < 0.05). Both CM and CSS were found to enhance the inner cell mass (ICM), trophectoderm (TE) and total cell numbers in blastocysts when used on both Days 1 and 3 (*p* < 0.05). Additionally, CM from Day 3 positively influenced the expression levels of development‐specific genes in cultured embryos (*p* < 0.05).

**Conclusion:**

Overall, the findings suggest that using bOEC‐CM at a 10% concentration may provide a promising supplement even better than serum and traditional co‐culture methods during the last 5 days of embryo culture.

## Graphical abstract

The study investigated the effects of bOEC‐CM on bovine in vitro embryo development. CM from Day 3 enhances blastocyst rate and positively affects the ICM and TE in blastocysts and increases the expression of development‐specific genes. bOEC‐CM may serve as a superior supplement during the final five embryo culture days.

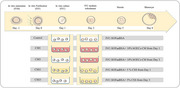

## 1 Introduction

Laboratory production of embryos has greatly advanced worldwide, primarily due to the acceleration of eugenics and the desire to increase the number of offspring with genetically distinct characteristics. In this regard, the quality of produced embryos and subsequently the implantation, pregnancy, and live birth rates are strongly affected by the culture medium (Coy, Romar, and Romero‐Aguirregomezcorta 2022). Today, significant progress has been made in developing optimal embryo culture media (Ferré et al. 2020). Nevertheless, the quality of embryos produced in vitro is still lower than that of in vivo counterparts (Mahdavinezhad et al. 2019) which may be attributed to the factors secreted from the oviduct. The pre‐implantation embryo is housed inside the oviduct, exposed to oviductal secretory fluid and epithelial cells (Li and Winuthayanon 2017; Banliat et al. 2022).

Laboratory systems are valuable tools for studying pathways and mechanisms that are difficult to investigate in vivo. In this regard, cell cultures provide valuable insights into physiological and pathological mechanisms. Somatic cells chosen for co‐culture should support embryogenesis and be compatible with the growth and development of embryos. These cells provide factors for the embryo to pass through the maternal‐zygotic stage (MZT) and complete its development (Ménézo et al. 2012; Maillo et al. 2016). The secreted cytokines and growth factors from somatic cells increase metabolic activity, subsequently enhancing the development and implantation ability of the embryo (Mohshina and Gedik 2021). Oviductal epithelial cells as nutrient cells secrete various cytokines and growth factors, such as epidermal growth factor (EGF), kit ligand, colony‐stimulating factor 1 (CSF‐1), leukaemia inhibitory factor (LIF) and interleukin‐6 (IL‐6) (Barmat, Worrilow, and Paynton 1997). These secreted factors exhibited a positive correlation with the development and quality of the embryo. Therefore, a co‐culture system of embryos with uterine epithelial cells leads to an increase in the secretion of embryonic paracrine markers (De los Santos et al. 1996), secretion of nutrients required by the embryos, such as growth factors, and the improvement of uterine receptivity by upregulating adhesion molecules (Lakshmi et al. 2022).

Despite the positive effects of co‐culture systems, there are problems in using these systems, including the complexity of the work method, the lack of reproducibility and the possibility of biological contamination (Ménézo et al. 2012). Considering the mentioned problems and in order to avoid direct contact between cells, such as bovine oviductal epithelial cell (bOEC) and embryo, other methods can be used instead of co‐culture, such as the use of conditioned media, which has positive effects on embryo development and survival rate after transfer (Lakshmi et al. 2022). Studies have shown that the conditioned medium (CM) of uterine epithelial cells is able to support embryo development to the blastocyst stage (Lakshmi et al. 2022) due to the presence of specific secreted nutrient compounds such as OVGP1 (Briton‐Jones et al. 2004), ET‐1 (Reinhart et al. 2003), insulin‐like growth factor (IGF), vascular endothelial growth factor (VEGF), epidermal growth factor (EGF), insulin‐like growth factor 1 (IGF1), transforming growth factor beta 1 (TGFβ2) and interlukin 4 (IL4) (Okada et al. 2005). Therefore, in this study, we attempted to determine the effect of using bOEC‐derived CM from the first and/or third days of embryo culture on subsequent embryo development, specifically the blastocyst production rate and the quality of the embryos.

## 2 Materials and Methods

### 2.1 Preparation of CM of bOECs

In this study, one bovine uterus was selected from slaughtered cattle that had recently ovulated. The selected uterus was transported to the laboratory in sterile normal saline supplemented with antibiotics (100 mg/mL streptomycin and 0.1 IU/mL penicillin) within less than 3 h at 30°C. The ipsilateral oviduct was carefully excised from the surrounding tissues, rinsed with physiological serum and disinfected with 70% ethanol. The isthmus end of oviduct was placed in a 1.5 mL microtube. The 0.5 mL of trypsin‐EDTA solution was then injected into the oviduct through the side of the ampulla, which was held in place with forceps. After 5 min, the oviduct was gently squeezed in a stripping motion with forceps along the ampulla and isthmus to obtain bOECs. After centrifugation at 500×*g* for 5 min, the isolated cells were cultured in bicabonate‐TCM199 medium containing 20% fetal calf serum (FCS) for 48 h in a 5% CO_2_ incubator at 38.5°C.

After reaching 70% confluency of cultured cells, the CM was obtained from the primary culture following the methods described in our previous studies (Nazifi et al. 2022; Shadmanesh et al. 2022). In brief, the cells were washed with PBS to remove any residual factors and serum and then re‐fed with serum‐free b‐TCM199 for 72 h. Finally, the CM was collected and centrifuged twice: first at 1000 g for 5 min at 4°C, and then at 4000 g for 15 min at 14°C. The final volume of prepared CM was 10 mL which was finally filtered through a 0.22 µm filter to remove cellular debris, aliquoted and stored frozen at −30°C until needed.

### 2.2 In Vitro Production of Embryo

Bovine embryos were produced in vitro according to the procedure commonly used in our laboratory (Nazifi et al. 2022; Shadmanesh et al. 2022). After collection of bovine ovaries at a local slaughterhouse and transfer to the laboratory, the immature cumulus‐oocyte complexes (COCs) were aspirated using an 18‐gauge needle connected to a 10‐mL syringe from ovarian follicles with a diameter of 2–8 mm. The COCs were washed four times in pre‐incubated HEPES‐TCM199 + 10% FBS droplets before being transferred to the maturation medium. The COCs were then matured into 50 µL in vitro maturation (IVM) medium drops, which included b‐TCM199 supplemented with 10% FBS, 0.05 mg/mL FSH (Folltropin; Vetoquinol), 5 mg/mL penicillin and 4 mg/mL streptomycin in a 9% CO_2_ incubator at 38.5°C for 24 h. The in vitro fertilization (IVF) of the matured oocytes was carried out with epididymal sperm into 50 µL drops of Tyrode's–albumin–lactate–pyruvate (TALP) medium containing 25 mg/mL heparin, 6 mg/mL bovine serum albumin (BSA), 5 mg/mL penicillin and 4 mg/mL streptomycin under the same conditions as IVM. A period of 22–24 h after the beginning of fertilization, presumptive zygotes were mechanically denuded of their cumulus cells and cultured into 20 µL drops of synthetic oviductal fluid (SOF) supplemented with amino acids, BSA (IVC‐SOFaaBSA) and Charcoled Strip Serum (CSS) or bOEC‐CM according to the experimental design under mineral oil in 9% CO_2_ and 7% O_2_, with a maximally humidified atmosphere. The refreshment of the culture medium for cleaved embryos was performed on the third day of culture (with the day of fertilization considered as Day 0).

### 2.3 Experimental Design

**FIGURE 1:**
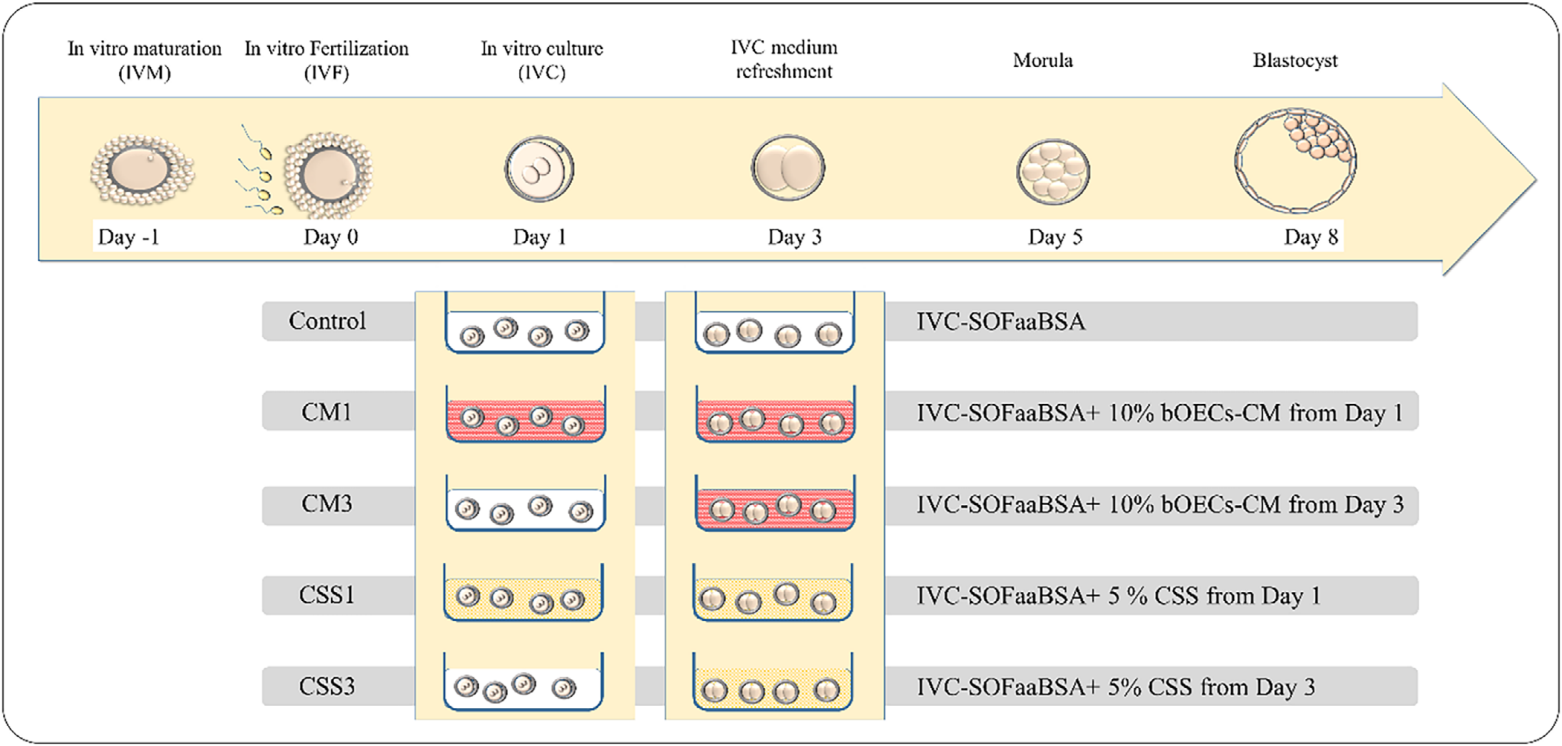
The experimental groups studied according the time of treatment with CSS or CM. The culture medium was IVC‐SOFaa + 8 mg/mL BSA. bOECs, bovine oviductal epithelial cells; CM, conditioned medium; CSS, charcoaled strip serum.

This study was designed as a randomized complete block to eliminate the effects of differences in embryonic quality between different replications (five replicates in each group). The embryos produced in vitro were treated with CSS or bOECs‐CM from Days 1 to 3 after fertilization in five groups as determined in Figure 1. The concentration of CSS was determined on the basis of the routine IVF procedure in the lab, whereas the concentration of CM was established after a pilot study involving concentrations of 5%, 10% and 15%.

During the experiment, the cleavage rate (/cultured COCs), blastocyst rate on Days7, 8 and 9 (/cleaved embryos) and hatching rate (/blastocyst Number) were ass. Quality of produced blastocysts on Day 8 in different groups was evaluated with both methods of differential cell staining, on 42 blastocysts (differential cell counting: inner cell mass ‘ICM’ trophectoderm cells ‘TE’), and the relative expression of OCT4, IFNt, COX2, PLAC8, BAX and Bcl2 genes, which were normalized with GAPDH as internal control reference gene (5 blastocysts in each replicate; totally 25 blastocysts per treatment).

### 2.4 Embryo Quality Assessment

#### 2.4.1 Differential Staining

Differential staining of ICM and TE compartments was carried out according to our previous studies (Shirazi et al. 2009; Shadmanesh et al. 2022). In brief, the 8‐day‐old expanding and expanded blastocysts were made permeable with 0.5% Triton X‐ 100 solution for 10 s and then were stained with 30 µg/mL Propidium Iodide (PI) solution for 1 min. The base medium of these solutions was H‐SOF containing 5 mg/mL BSA. After washing in base medium, the blastocysts were transferred in ice‐cold ethanol containing 10 µg/mL Hoechst 33,342, for 5 min. The blastocysts were then mounted into the small droplet of glycerol on glass slide and examined under an epi‐fluorescent microscope (Olympus IX71; Olympus). The ICM and TE cells appeared blue and violet (blue‐red), respectively, due to a DNA labelling with Hoechst 33,342 as a membrane permeable fluorescent dye, and PI as a membrane impermeable fluorescent dye.

#### 2.4.2 Molecular Evaluation

To determine the relative expression of desired genes, a pool of five 8‐day‐old blastocysts was stored for each replication in 10 µL water in a 0.25 mm peyote in liquid nitrogen until RNA extraction. Total RNA was extracted using Totla RNA extraction kit (viragene, Tehran, Iran) as a column based extraction protocol according to the manufacturer's instruction. Briefly, samples were lysed with 0.5 mL cell lysis buffer and incubated at 65°C for 5 min. After centrifugation and transfer the supernatant to another 1.5 mL microtube, the equivalent volume (0.5 mL) of isopropanol was added. The mixed solution was transferred to a microtube containing the column, centrifuged and washed the column twice. Finally, the binded RNA to the spin column was soluted in elution buffer solution and then treated by DNase (Sinaclon Bioscience) for remove eventual residual DNA. Total RNA was measured and quantified Spectrophotometrically.

RNA was reverse‐transcribed into cDNA using Yektatajhiz‐Azma cDNA Synthesis Kit (Tehran, Iran; Cat No: YT4500). The obtained cDNA was stored at −30°C.

The levels of OCT4, IFNt, COX2, PLAC8, BAX and Bcl2 transcripts were determined by real‐time reverse transcriptase (RT)‐PCR using RealQ Plus 2x Master Mix Green (Amplicon, Denmark). GAPDH was used as an endogenous standard to normalize the input load of cDNA among samples, as introduced previously (Goossens et al. 2005). Specific primers were designed with Primer‐Blast (https://www.ncbi.nlm.nih.gov/tools/primer‐blast); details are presented in Table 1. The PCR was carried out in a real‐time PCR cycler (Rotor Gene Q 6000; Qiagen, Valencia, CA, USA) in two replicates for each sample. The thermal profile was 95°C for 15 min, 40 cycles of 94°C for 15 s, 59°C–62°C for 20 s (annealing temperature; Table 1) and 72°C for 25 s.

**TABLE 1:** Sequence and annealing temperature of primers used for real time reverse transcriptase (RT)‐PCR.

Gene	Gene bank accession no.	Primer sequence (5′‐3′)	Product size (bp)	Annealing temp. (°C)
*GAPDH*	NM_001034034.2	F‐GATGGTGAAGGTCGGAGTGAAC	100	62
		R‐GTCATTGATGGCGACGATGT		
*Pou5f1* (*Oct 4*)	NM_174580.2	F‐TGGACAAGGAGAAGCTGGAG	130	59
		R‐ACATCGGCCTGGGTATATCC		
*IFNt*	NM_001015511.4	F‐CTGGCCCGAATGAACAGACT	102	62.5
		R‐ATCCTTCTGGAGCTGGTTGC		
*PLAC8*	NM_00102535.2	F‐CTGCTGATCTGAGCTGCTGT	107	60
		R‐GAGCTGTAACCTGGCTGTGA		
*COX2*	NM_174445.2	F‐TCTACCCGCCTCATGTTCCT	112	62.5
		R‐CTGTTGTGTTCCCGTAGCCA		
*BAX*	NM_173894.1	F‐GTGCCCGAGTTGATCAGGAC	110	60
		R‐AAGTAGGAGAGGAGGCCGT		
*BCL2*	NM_001166486.1	F‐CTTCGCCGAGATGTCCAGTC	95	60
		R‐TCACCCCGTCCCTGAAGA		

To give the efficiency of PCR reaction, data were analysed using LINREGPCR software (version 2012.0, LinRegPCR) (Ruijter et al. 2009). Relative abundance of transcripts was calculated using efficiency adjusted Paffl methodology (Dorak 2007).

$$\mathrm{Ratio}=\frac{E_{\mathrm{GAPDH}}\left(C_{t}\mathrm{Treatment}\right)/E_{\mathrm{target}}\left(C_{t}\mathrm{Treatment}\right)}{E_{\mathrm{GAPDH}}\left(C_{t}\mathrm{Control}\right)/E_{\mathrm{target}}\left(C_{t}\mathrm{Control}\right)}$$

### 2.5 Statistical Analysis

All data were normally distributed and tested for homogeneity of variance by Shapiro–Wilk test and other tests. The data related to embryo development (cleavage, blastocyst, and hatching rates) and differential staining and relative genes expression were analysed using one‐way ANOVA followed by Tukey post hoc test. Statistical analysis was performed using IBM SPSS 25 software package. The results were reported as Mean ± SD, and differences were considered significant at the level of *p* < 0.05.

## 3 Results

As the treatment in CSS3 and CM3 groups was started from the Day 3, the cleavage rate was measurable only in the control, CSS1 and CM1 groups, which has been shown in Table 2.

**TABLE 2:** The cleavage rate of bovine presumptive zygotes in different culture conditions.

Treatments	Oocyte No.	Cleaved embryos No. (% ± SEM)
Control	374	246 (74.11 ± 1.12)^ab^
CM1	328	226 (69.42 ± 1.97)^b^
CSS1	290	229 (78.98 ± 2.97)^a^

*Note*: ^a,b^ Different superscripts in the same column denote a significant difference (*p *< 0.05).

Abbreviations: CSS, Charcoled Strip Serum (Used in 5% concentration); CM, Conditioned Medium (Used in 10% concentration).

The addition of 10% CM from Day 1 (CM1) to the embryos culture medium led to a significant decrease in the cleavage rate when compared to use of CSS in the same time point (CSS1) (*p* = 0.015). Furthermore, neither CSS nor CM exhibited a significant effect on cleavage rate in comparison to the control group.

In comparison to the control group, CSS increased the blastocyst rate on Days 7, 8 and 9 (*p* < 0.05); conversely, the CM has a significant effect only when used from Day 3 (*p* < 0.05), and this positive effect was significantly higher than CSS (*p* < 0.05). Considering the earlier blastocyst production in CSS groups, it can be said that CSS enhanced the development speed compared to the CM, as a significant number of blastocysts in the CM groups were observed on Day 9. Furthermore, the addition of 10% CM on Day 3 significantly improved the hatching rate compared to all other groups (*p* < 0.05), indicating superior quality of the embryos produced in this group (Table 3).

**TABLE 3:** The blastocyst and hatching rate of bovine embryos in different culture conditions.

Treatment	Cleaved embryos no.	Blastocyst rate/cleaved embryos (% ± SEM)			Hatching rate/blastocyst no. (% ± SEM)
		Day 7	Day 8	Day 9	
Control	246	8.49 ± 1.63a	15.17 ± 2.21a	17.44 ± 2.09a	6.69 ± 3.07a
CM1	226	12.58 ± 1.68ac	17.31 ± 1.92ac	22.19 ± 1.81ab	11.12 ± 6.53b
CM3	245	18.83 ± 2.46b	29.39 ± 2.23b	34.59 ± 2.45d	39.03 ± 6.16d
CSS1	229	17.34 ± 3.00bc	22.95 ± 3.24bc	25.09 ± 3.19bc	16.78 ± 5.49b
CSS3	242	21.22 ± 0.88b	28.08 ± 1.71b	29.48 ± 1.30cd	28.58 ± 6.22c

*Note*: ^a,b^ Different superscripts in the same column denote a significant difference (*p* < 0.05).

Blastocyst staining analysis indicated that CM and CSS increase the ICM, TE and total cells numbers in Day 8 blastocysts following administration on Days 1 and 3, in comparison to the control group (*p* < 0.05). No significant differences were observed in the ICM and TE cells numbers among blastocysts cultured in presence of CSS and CM (CSS1, CSS3, CM1 and CM3 groups). However, the total cells number was significantly higher in embryos treated with CM from Day 3 (CM3 group) at the blastocyst stage, when compared to the other groups (*p* < 0.05). Additionally, no significant differences in the ratio of ICM/total cells number were observed among the groups (Table 4).

**TABLE 4:** The cell numbers of in vitro produced bovine blastocysts in different culture conditions.

Treatment	Blastocyst No.	Blastocyst cells (mean ± SEM)			ICM/Total
		ICM	TE	Total	
Control	7	16.42 ± 2.13a	38.43 ± 1.48a	54.86 ± 3.37a	0.29 ± 0.02
CM1	8	27.87 ± 1.62bc	55.75 ± 2.72b	83.68 ± 3.92b	0.33 ± 0.01
CM3	9	33.22 ± 1.22b	64.55 ± 3.67b	97.78 ± 4.40c	0.35 ± 0.02
CSS1	8	26.5 ± 1.59c	52.75 ± 2.71b	79.25 ± 3.12b	0.34 ± 0.01
CSS3	9	27.59 ± 1.96bc	52.78 ± 3.42b	80.33 ± 5.13b	0.34 ± 0.01

*Note*: ^a,b,c^ Different superscripts in the same column denote a significant difference (*p* < 0.05).

Abbreviations: ICM, inner cell mass; TE, trophectoderm.

**FIGURE 2:**
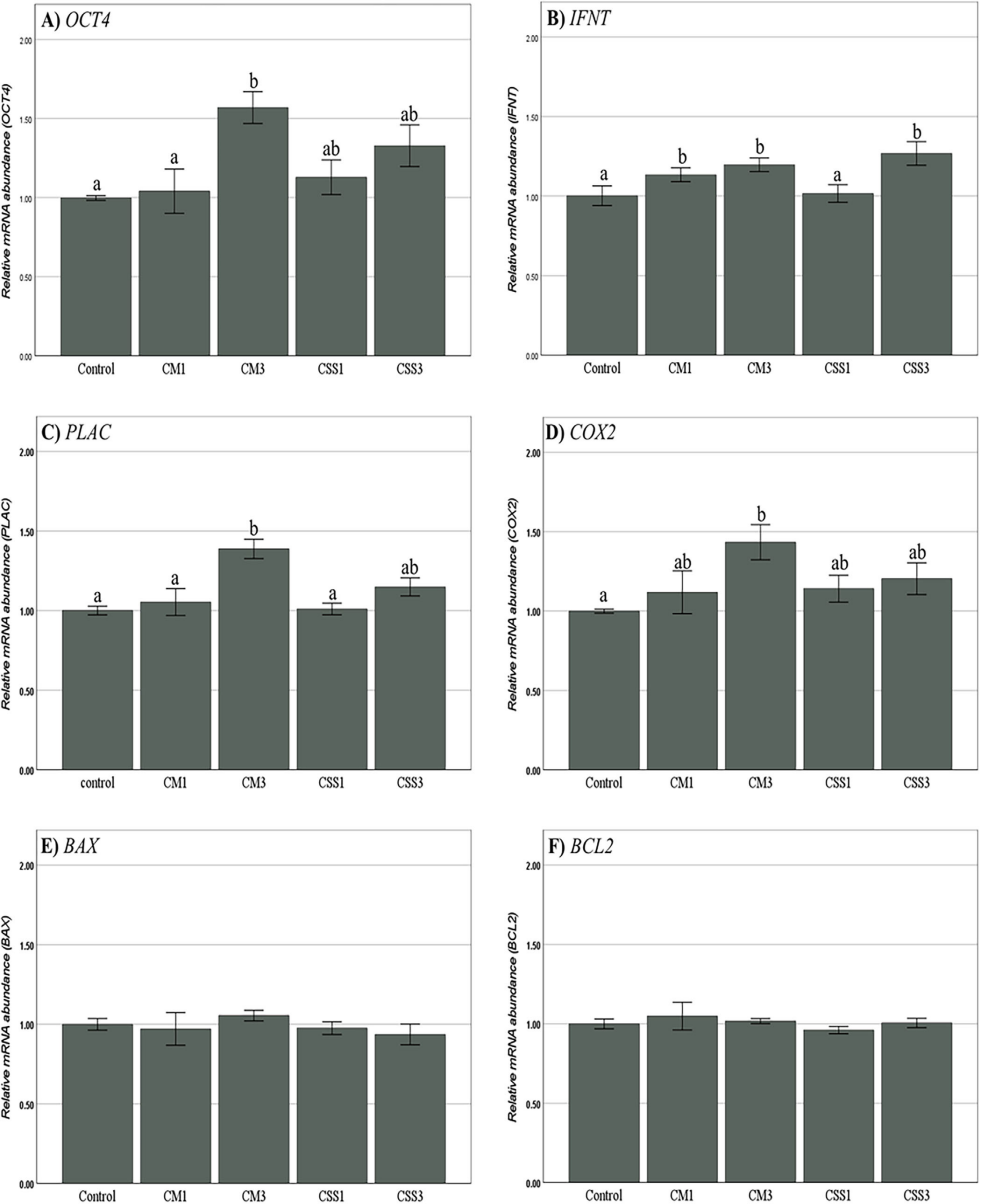
Relative abundance of *OCT4* (A), *IFNT* (B), *PLAC* (C), *COX2* (D), *BAX* (E) and *BCL2* (F) transcripts in bovine blastocyst derived from different culture conditions. The mRNA from pools of 5 blastocysts was reverse transcribed and subjected to real‐time quantitative PCR. All reactions were normalized for *GAPDH* mRNA expression. Values with superscripts “a,b” refers to significant (*p* < 0.05) differences in relative transcript abundance between groups.

The expression level of Oct4 in embryos of CM3 group was significantly higher than that observed in the embryos of control and CM1 groups (*p* < 0.05). The addition of CSS did not result in a significant effect on the expression level of this gene when compared to the control group (Figure 2A).

The expression of IFNT in embryos treated with 10% CM (CM1 and CM3) exhibited a significant increase compared to the control group (*p* < 0.05). This effect was not observed when using CSS (Figure 2B).

The expression level of PLAC8 in embryos treated with 10% CM from Day 3 (CM3) was significantly higher than the control, CSS1 and CM1 groups (*p* < 0.05). No significant differences in the expression of this gene were observed among the other groups (Figure 2C).

COX2 was significantly elevated in the CM3‐10% group when compared to the control group (*p* < 0.05). No significant difference was observed among the other groups (Figure 2D).

In this study, no significant differences were observed in the expression levels of BAX (Figure 2E) and BCL2 (Figure 2F) genes. These findings indicate that the use of CM and serum (CSS) does not significantly affect the expression of apoptosis‐related genes (*p* < 0.05).

## 4 Discussion

The results of this study indicated that the use of CM obtained from bOECs on the third day of culture (CM3) had a notable influence on the blastocyst rate. Particularly, the CSS accelerated the developmental speed of produced embryos when compared to the control group. Additionally, a significant disparity in the hatching rate was observed between the CM3 group and the control group and other experimental groups, suggesting the efficacy of the CM in improving the quality of the resulting blastocysts.

The absence of a significant impact observed when utilizing CM from the first day (CM1) may be attributed to variances in embryo metabolism between early embryonic stages and more advanced stages. Embryos at early stages have unique nutrient requirements and display distinct metabolic activities. These results contrast with those of Cordova et al. (2014), who utilized bOECs in co‐culture with embryos at early (Days 1–4) or advanced (Days 4–7) stages, showing that the presence of bOECs during the initial 4 days of embryo development, resembling the oviductal secretions in in vivo environment, accelerates blastocyst development kinetics. It is important to note that the differing outcomes between our study and Cordova's study may be attributed to the use of CM in our research, as opposed to the co‐culture system utilized in their investigation. Many studies have indicated that FBS does not significantly influence the cleavage rate. However, the presence of FBS in the culture media has been associated with an increased rate of development to the blastocyst stage. Furthermore, embryos cultured without FBS exhibited delays in their developmental progression (Han and Niwa 2003; Arnold et al. 2015), which aligns with the findings of our study.

The observed lower cleavage rates in presumptive zygotes culture in presence of CM can be attributed to the use of TCM199 as the basic medium for the CM preparation. It has been established that the 2‐ to 4‐cell embryos require oxidative phosphorylation, whereas morulae and blastocysts do not. Consequently, the presence of phosphate and glucose, which are two components of M199, may interfere with the oxidative phosphorylation processes occurring in embryos as they develop from the 8‐cell stage to the blastocyst stage (Seshagiri and Bavister 1991; Brison and Leese 1994; Han and Niwa 2003). In addressing the ineffectiveness of the base medium of CM(TCM199) on the development of cultured embryos, it is important to consider that the more complex TCM199 medium was designed primarily for the maintenance of somatic cells in vitro, rather than for the culture of early mammalian embryos. It has been suggested that bovine embryos produced using the SOF system exhibit greater similarities to their in vivo counterparts, particularly in terms of gene expression patterns (Wrenzycki et al. 2001; Yaseen et al. 2001), compared to those generated via the TCM199 system. Additionally, the reduced oxygen tension present in the SOF system may mitigate the detrimental effects of reactive oxygen species (ROS), aligning more closely with the oviductal microenvironment (Gordon 2003). Consequently, it can be inferred that the beneficial effects of the bOEC‐CM are attributable not to the TCM199 medium utilized in CM preparation, but rather to the factors secreted by oviductal epithelial cells (OECs) that are present in the CM and accessible to the bovine embryo.

A study conducted on the impact of embryo interaction with CM derived from bOEC cells demonstrated an increase in the percentage of cleavage and blastocyst formation (Fukui et al. 1991). Another studies clearly indicated that oviduct epithelial cells can create a favourable environment for embryo development (Yeung et al. 1992; Cordova et al. 2014; Bastos et al. 2022). Additionally, it was found that the percentage of blastocysts produced in CM was higher compared to co‐culture and M199 medium containing FCS (Eyestone and First 1989). Furthermore, bOECs were found to secrete embryonic factors, such as Complement Component C3, Oviductin and Osteopontin, in the CM, which were shown to regulate and enhance embryo growth (Lee et al. 2004; Gonçalves, Wolinetz, and Killian 2007; Hao et al. 2008). Other studies investigated the effect of growth factors secreted by OECs, including TGF‐a, TGF‐b, IGF‐1, IGF‐2, FGF‐2 and EGF, on the development of embryo to morula and blastocyst stages in the OECs‐CM in vitro, yielding positive outcomes (Kane, Morgan, and Coonan 1997; Jung et al. 2011; Schmaltz‐Panneau et al. 2015; Fang et al. 2022).

IGF‐1 has been shown to stimulate cell proliferation in pre‐implantation embryos, protect them against heat shock, and also act as an anti‐apoptotic factor (Jousan and Hansen 2007). In a study, the addition of a combination of bFGF, GM‐CSF and LIF to the mSOF medium significantly increased the percentage of blastocysts on day 8 after fertilization compared to using the mSOF medium alone (Neira et al. 2010). Therefore, the presence of these growth factors and cytokines in the CM may have a stimulating and supportive effect on embryo growth in vitro. The financial limitations inherent to this study precluded a comprehensive analysis of the components of the CM. However, the results obtained, which align closely with those from previous studies that have examined the components of conditioned media, imply the likely presence of these factors within the CM employed in current study.

Many studies have been carried out to explore the efficacy of CM derived from various cell lines. For instance, Park et al. (2013) conducted a study in which they utilized 10% CM derived from human mesenchymal stem cells (MSC) to culture porcine embryos in vitro. Additionally, 10% CM obtained from embryonic stem cells was employed to culture bovine embryos in another study (Uhm 2023), and the bovine amniotic membrane stem cell‐CM (bAMSC‐CM) was utilized to culture ovine embryos in another study (Nikoobin et al. 2024). In these studies, the authors observed enhancements in the growth and development of embryos. Notably, the best results were observed when the CM groups were compared with the control that was FCS free. Furthermore, they did not observed any significant difference in blastocyst rate between the use of 10% FCS and 10% CM, which is consistent with the results of the present study.

Conditioned media contain growth factors and binding proteins (BP) that are secreted by cells. These factors are present in both soluble form and in microvesicles (MVs) (Bhardwaj et al. 2016). MVs are composed of proteins, lipids (specifically sphingomyelins in high quantities), and various types of RNAs, including microRNAs (miRNAs) and mRNA fragments (Simons and Raposo 2009; da Silveira et al. 2012; Raposo and Stoorvogel 2013). Research has shown that MVs are actively secreted by most cells and can be found in various organic fluids, including fallopian tube fluids during the reproductive cycle (Burns et al. 2014, Machtinger, Laurent, and Baccarelli 2016; Perrini et al. 2018). Adding extracellular fluid vesicles from the oviduct tube to FCS‐free culture medium has been found to improve the growth and quality of embryos (Lopera‐Vasquez et al. 2017). MVs play a crucial role in facilitating the interaction between the embryo and its surrounding microenvironment, which is influenced by the endometrium in vivo and by different culture conditions in vitro (Machtinger, Laurent, and Baccarelli 2016).

The positive effects of OECs on the growth and development of parthenogenetic and cloned porcine pre‐implantation embryos have been attributed to the presence of extracellular vesicles. These vesicles play role in various improvement mechanisms, including the transfer of mRNA transcripts, antioxidant defence, protection against cell apoptosis and increased expression of pluripotency genes. Extracellular vesicles function as communication nanoparticles between cells and contain miRNAs and small RNA molecules that regulate cell signalling and epigenetics. Studies have shown that extracellular vesicles derived from the fallopian tube can interact with oocytes and deliver their molecular contents into the gamete cytoplasm. Additionally, supplementation of these extracellular vesicles reduces levels of ROS and increases the expression of POU5F1, NANOG, SOX2 and GATA6 genes in embryos.

One way to evaluate the quality of embryos is by examining the expression of genes related to development. Any deviation in the expression of these genes during in vitro culture can lead to disturbance in producing high‐quality blastocysts (Chason et al. 2011). In this study, the expression of four genes (OCT4, IFNT, PLAC8 and COX2) involved in the development of pre‐implantation embryos, as well as two genes (BAX and BCL2) related to embryo apoptosis, were investigated. Previous studies have focused on analysing the expression of OCT4, a key gene in pre‐implantation development. OCT4 is a crucial regulator of pluripotent cells in mammals. It promotes the proliferation of ICM and TE cells in the blastocyst by stimulating the secretion of IGF (Kirchhof et al. 2000; Tamrin et al. 2020). In this study, the expression of OCT4 was found to be higher in the embryos of the CM3 group compared to the control and CM1 groups. The addition of CSS did not have a significant effect on the expression of this gene compared to the control group. These findings align with previous researches on the relationship between OCT4 expression, embryo development rate, and quality (Kim et al. 2011; Lee et al. 2015; Sakurai et al. 2016; Tamrin et al. 2020). In the study conducted by Shadmanesh et al., the expression of the OCT4 gene was found to be significantly increased in bovine embryos when cultured in presence of CM derived from human amniotic membrane stem cells (hAMSCs‐CM).

Previous research has reported the expression of the PLAC8 gene in various species, including zebrafish, mouse (Galaviz‐Hernandez et al. 2003), bovine (El‐Sayed et al. 2006), rabbit and human (Li et al. 2016), during the morula and blastocyst stages. Yan et al. (2013) demonstrated that PLAC8 expression in human embryos increases from the one‐cell stage to the blastocyst stage. This gene appears to play a potential role in placental development and the interaction between the foetus and the mother, and its expression level is indicative of embryo quality. In the present study, the expression level of the PLAC8 gene was significantly higher in the CM3 group compared to the control, CSS1 and CM1 groups. However, this significant difference was not observed when compared to the CSS3 group. The high level of PLAC8 expression was directly correlated with the blastocyst rate and embryo quality and consistent with other studies (El‐Sayed et al. 2006; Ghanem et al. 2011; Li et al. 2016; Tamrin et al. 2020). For example, research on bovine embryos at the blastocyst stage has shown that blastocysts with higher PLAC8 expression are more likely to result in live births (El‐Sayed et al. 2006). Additionally, in Shadmanesh et al. study, the expression level of the PLAC8 gene during the pre‐implantation stage of bovine embryo had a direct relationship with the use of CM.

Prostaglandins and derivatives of arachidonic acid play a crucial role in pre‐implantation embryo development. Cyclooxygenase (COX), an important enzyme involved in prostaglandin biosynthesis, acts as an oxygenase and peroxidase (Salleh 2014). COX2 is secreted from the TE cells of the blastocyst during the pre‐implantation stage and is necessary for blastocyst hatching. This factor is also associated with PGI2 and PGE2, which are important for embryo development. Therefore, the expression level of COX2 can serve as an indicator of blastocyst quality (Ghanem et al. 2011). In this study, the expression of the COX2 gene was significantly higher in the CM3 group compared to the control group, which is consistent with the high blastocyst and hatching rates observed in the CM3 group. This finding suggests that the higher expression of COX2 can be a positive indicator of embryo quality and is consistent with results from other studies that have confirmed the direct relationship between the expression level of this gene and the developmental rate of resulting embryos (Wang et al. 2002; Ghanem et al. 2011; Salleh 2014). Assessment of COX2 gene expression in bovine embryos cultured in the presence of hAMSCs‐CM in the study by Shadmanesh et al. confirmed the positive effect of CM on the development of bovine embryos, which aligns with the findings of the present study.

The assessment of IFNT gene expression levels revealed a significant increase in embryos treated with CM from the first or third days (CM1 and CM3 groups) compared to the control group, whereas no such effect was observed with CSS. This finding aligns with previous research indicating that embryos regulate the expression of immune response‐related genes by upregulating interferon‐related genes when exposed to CM from mare oviducts (Smits et al. 2016). Recent studies have also suggested that bOECs stimulate embryos to produce IFNT, potentially influencing immune cells to elicit an anti‐inflammatory response in the oviduct (Talukder et al. 2018). Factors such as FGF2 and EGF have been shown to enhance IFNT secretion by foetal trophoblast cells during pregnancy, underscoring the role of oviduct‐secreted factors in IFNT expression and synthesis in pre‐implantation embryos.

In terms of apoptosis, it is commonly believed that in vitro conditions can potentially increase the rates of apoptosis in developing embryos. This study aimed to assess the expression levels of two key genes involved in apoptosis, namely, BAX and BCL2. However, no significant differences were observed in the expression of these genes, indicating that the use of CM and CSS did not notably impact the expression of apoptosis‐related genes.

## 5 Conclusion

In view of the ease of preparation and maintenance, as well as the significant impact observed on blastocyst rate, hatching rate, and embryo quality, the use of bOECs‐CM at a 10% concentration shows promise as a supplement even better than serum and maybe co‐culture methods for the final 5 days of embryo culture (third day's embryos). It is important to note that throughout this study, the conditioned media was consistently prepared and applied from multiple bovine oviducts, with varying effects on bovine embryo development, occasionally showing no discernible positive influence. The reasons for these inconsistencies remain unknown and require further in‐depth investigation.

## Author Contributions

Saeed Kazemi Hosseinabadi contributed to the planning and performance of the study; Hassan Nazari contributed to the planning and performance of the study, and writing and revising of the manuscript; Mehran Arabi contributed to the planning of the study, and revising the manuscript; Naser Shams Esfandabadi and Ebrahim Ahmadi contributed to the planning the study and statistical analysis; Azita Afzali and Hamidreza Ghanaei contributed to performance of the study.

## Ethics Statement

Animal husbandry and handling were conducted in accordance with the guidelines of the ethical committee of Shahrekord University, Iran.

## Conflicts of Interest

The authors declare no conflicts of interest

### Peer Review

The peer review history for this article is available at https://publons.com/publon/10.1002/vms3.70179.

## Data Availability

Data are available on request from the authors.

## References

[vms370179-bib-0001] Arnold, D. R. , C. A. P. Corrêa , L. L. G. Lorena , et al. 2015. “Supplementation of Fetal Bovine Serum Alters Histone Modification H3R26me2 During Preimplantation Development of In Vitro Produced Bovine Embryos.” Pesquisa Veterinária Brasileira 35, no. 7: 605–612. 10.1590/S0100-736x2015000700002.

[vms370179-bib-0002] Banliat, C. , C. Mahé , R. Lavigne , et al. 2022. “The Proteomic Analysis of Bovine Embryos Developed In Vivo or In Vitro Reveals the Contribution of the Maternal Environment to Early Embryo.” BMC Genomics [Electronic Resource] 23, no. 1: 839. 10.1186/s12864-022-09076-5. 36536309 PMC9764490

[vms370179-bib-0003] Barmat, L. I. , K. C. Worrilow , and B. V. Paynton . 1997. “Growth Factor Expression by Human Oviduct and Buffalo Rat Liver Coculture Cells.” Fertility and Sterility 67, no. 4: 775–779. 10.1016/s0015-0282(97)81382-9. 9093210

[vms370179-bib-0004] Bastos, N. M. , J. G. Ferst , R. S. Goulart , and J. Coelho da Silveira . 2022. “The Role of the Oviduct and Extracellular Vesicles During Early Embryo Development in Bovine.” Animal Reproduction 19, no. 1: 1–13. 10.1186/s40104-024-01008-5. PMC9037602 35493787

[vms370179-bib-0005] Bhardwaj, R. , M. M. Ansari , M. S. Parmar , V. Chandra , and G. T. Sharma . 2016. “Stem Cell Conditioned Media Contains Important Growth Factors and Improves In Vitro Buffalo Embryo Production.” Animal Biotechnology 27, no. 2: 118–125. 10.1080/10495398.2015.1118383. 26913553

[vms370179-bib-0006] Brison, D. R. , and H. J. Leese . 1994. “Blastocoel Cavity Formation by Preimplantation Rat Embryos in the Presence of Cyanide and Other Inhibitors of Oxidative Phosphorylation.” Journal of Reproduction and Fertility 101, no. 2: 305–309. 10.1530/jrf.0.1010305. 7932362

[vms370179-bib-0007] Briton‐Jones, C. , I. H. Lok , C. K. Cheung , T. T. Chiu , L. P. Cheung , and C. Haines . 2004. “Estradiol Regulation of Oviductin/Oviduct‐Specific Glycoprotein Messenger Ribonucleic Acid Expression in Human Oviduct Mucosal Cells In Vitro.” Fertility and Sterility 81, no. S1: 749–756. 10.1016/j.fertnstert.2003.08.016. 15019805

[vms370179-bib-0008] Burns, G. , K. Brooks , M. Wildung , R. Navakanitworakul , L. K. Christenson , and T. E. J. P. o. Spencer . 2014. “Extracellular Vesicles in Luminal Fluid of the Ovine Uterus.” PLoS ONE 9, no. 3: e90913. 10.1371/journal.pone.0090913. 24614226 PMC3948691

[vms370179-bib-0009] Chason, R. J. , J. Csokmay , J. H. Segars , A. H. DeCherney , and D. R. Armant . 2011. “Environmental and Epigenetic Effects Upon Preimplantation Embryo Metabolism and Development.” Trends in Endocrinology and Metabolism 22, no. 10: 412–420. 10.1016/j.tem.2011.05.005. 21741268 PMC3183171

[vms370179-bib-0010] Cordova, A. , C. Perreau , S. Uzbekova , C. Ponsart , Y. Locatelli , and P. Mermillod . 2014. “Development Rate and Gene Expression of IVP Bovine Embryos Cocultured With Bovine Oviduct Epithelial Cells at Early or Late Stage of Preimplantation Development.” Theriogenology 81, no. 9: 1163–1173. 10.1016/j.theriogenology.2014.01.012. 24629595

[vms370179-bib-0011] Coy, P. , R. Romar , and J. Romero‐Aguirregomezcorta . 2022. “The Embryo Culture media in the Era of Epigenetics: Is It Time to Go Back to Nature?” Animal Reproduction 19: e20210132. 10.1590/1984-3143-AR2021-0132. 35493788 PMC9037603

[vms370179-bib-0012] da Silveira, J. C. , D. N. Veeramachaneni , Q. A. Winger , E. M. Carnevale , and G. J. Bouma . 2012. “Cell‐Secreted Vesicles in Equine Ovarian Follicular Fluid Contain miRNAs and Proteins: A Possible New Form of Cell Communication Within the Ovarian Follicle.” Biology of Reproduction 86, no. 3: 71. 10.1095/biolreprod.111.093252. 22116803

[vms370179-bib-0013] De los Santos, M. J. , A. Mercader , A. Francés , et al. 1996. “Role of Endometrial Factors in Regulating Secretion of Components of the Immunoreactive Human Embryonic Interleukin‐1 System During Embryonic Development.” Biology of Reproduction 54, no. 3: 563–574. 10.1095/biolreprod54.3.563. 8835377

[vms370179-bib-0014] Dorak, M. T. 2007. “Real‐Time PCR,” *Taylor & Francis*. 10.4324/9780203967317.

[vms370179-bib-0015] El‐Sayed, A. , M. Hoelker , F. Rings , et al. 2006. “Large‐Scale Transcriptional Analysis of Bovine Embryo Biopsies in Relation to Pregnancy Success After Transfer to Recipients.” Physiological Genomics 28, no. 1: 84–96. 10.1152/physiolgenomics.00111.2006. 17018689

[vms370179-bib-0016] Eyestone, W. H. , and N. L. First . 1989. “Co‐Culture of Early Cattle Embryos to the Blastocyst Stage With Oviducal Tissue or in Conditioned Medium.” Journal of Reproduction and Fertility 85, no. 2: 715–720. 10.1530/jrf.0.0850715. 2704004

[vms370179-bib-0017] Fang, X. , B. M. Tanga , S. Bang , et al. 2022. “Oviduct Epithelial Cell‐Derived Extracellular Vesicles Improve Porcine Trophoblast Outgrowth.” Veterinary Science 9, no. 11: 609. 10.3390/vetsci9110609. PMC9697688 36356086

[vms370179-bib-0018] Ferré, L. , M. Kjelland , L. Strøbech , P. Hyttel , P. Mermillod , and P. Ross . 2020. “Recent Advances in Bovine in Vitro Embryo Production: Reproductive Biotechnology History and Methods.” Animal 14, no. 5: 991–1004. 10.1017/S1751731119002775. 31760966

[vms370179-bib-0019] Fukui, Y. , L. McGowan , R. James , P. Pugh , and H. Tervit . 1991. “Factors Affecting the In‐Vitro Development to Blastocysts of Bovine Oocytes Matured and Fertilized in Vitro.” Reproduction (Cambridge, England) 92, no. 1: 125–131. 10.1530/jrf.0.0920125. 1905351

[vms370179-bib-0020] Galaviz‐Hernandez, C. , C. Stagg , G. De Ridder , et al. 2003. “Plac8 and Plac9, Novel Placental‐Enriched Genes Identified Through Microarray Analysis.” Gene 309, no. 2: 81–89. 10.1016/s0378-1119(03)00508-0. 12758124

[vms370179-bib-0021] Ghanem, N. , D. Salilew‐Wondim , A. Gad , et al. 2011. “Bovine Blastocysts With Developmental Competence to Term Share Similar Expression of Developmentally Important Genes Although Derived From Different Culture Environments.” Reproduction (Cambridge, England) 142, no. 4: 551–564. 10.1530/REP-10-0476. 21799070

[vms370179-bib-0022] Gonçalves, R. F. , C. D. Wolinetz , and G. J. Killian . 2007. “Influence of Arginine‐Glycine‐Aspartic Acid (RGD), Integrins (AlphaV and Alpha5) and Osteopontin on Bovine Sperm‐Egg Binding, and Fertilization In Vitro.” Theriogenology 67, no. 3: 468–474. 10.1016/j.theriogenology.2006.08.013. 17030360

[vms370179-bib-0023] Goossens, K. , M. Van Poucke , A. Van Soom , J. Vandesompele , A. Van Zeveren , and L. J. Peelman . 2005. “Selection of Reference Genes for Quantitative Real‐Time PCR in Bovine Preimplantation Embryos.” BMC Developmental Biology 5: 27–35. 10.1186/1471-213x-5-27. 16324220 PMC1315359

[vms370179-bib-0024] Gordon, I. 2003. "Laboratory Production Of Cattle Embryos", *CABI* 2nd Edition.

[vms370179-bib-0025] Han, M. S. , and K. Niwa . 2003. “Effects of BSA and Fetal Bovine Serum in Culture Medium on Development of Rat Embryos.” Journal of Reproduction and Development 49, no. 3: 235–242. 10.1262/jrd.49.235. 14967933

[vms370179-bib-0026] Hao, Y. , C. N. Murphy , L. Spate , et al. 2008. “Osteopontin Improves in Vitro Development of Porcine Embryos and Decreases Apoptosis.” Molecular Reproduction and Development 75, no. 2: 291–298. 10.1002/mrd.20794. 17874454

[vms370179-bib-0027] Jousan, F. D. , and P. J. Hansen . 2007. “Insulin‐Like Growth Factor‐I Promotes Resistance of Bovine Preimplantation Embryos to Heat Shock Through Actions Independent of Its Anti‐Apoptotic Actions Requiring PI3K Signaling.” Molecular Reproduction and Development 74, no. 2: 189–196. 10.1002/mrd.20527. 16955404

[vms370179-bib-0028] Jung, J. G. , T. S. Park , J. N. Kim , et al. 2011. “Characterization and Application of Oviductal Epithelial Cells In Vitro in Gallus domesticus1.” Biology of Reproduction 85, no. 4: 798–807. 10.1095/biolreprod.111.092023. 21715713

[vms370179-bib-0029] Kane, M. T. , P. M. Morgan , and C. Coonan . 1997. “Peptide Growth Factors and Preimplantation Development.” Human Reproduction Update 3, no. 2: 137–157. 10.1093/humupd/3.2.137. 9286738

[vms370179-bib-0030] Kim, E. Y. , J. B. Lee , H. Y. Park , C. J. Jeong , K. Z. Riu , and S. P. Park . 2011. “The Use of Embryonic Stem Cell Derived Bioactive Material as a New Protein Supplement for the In Vitro Culture of Bovine Embryos.” Journal of Reproduction and Development 57, no. 3: 346–354. 10.1262/jrd.10-113a. 21289468

[vms370179-bib-0031] Kirchhof, N. , J. Carnwath , E. Lemme , K. Anastassiadis , H. Scholer , and H. Niemann . 2000. “Expression Pattern of Oct‐4 in Preimplantation Embryos of Different Species.” Biology of Reproduction 63, no. 6: 1698–1705. 10.1095/biolreprod63.6.1698. 11090438

[vms370179-bib-0032] Lakshmi, D. H. , N. D. Shital , P. Shriti , T. Yasotha , C. Vikash , and S. G. Taru . 2022. “Impact of Uterine Epithelial Cells and Its Conditioned Medium on the in Vitro Embryo Production in Buffalo (*Bubalus bubalis*).” Theriogenology 183: 61–68. 10.1016/j.theriogenology.2022.02.016. 35219005

[vms370179-bib-0033] Lee, S.‐E. , J. J.‐M. Moon , E.‐Y. Kim , and S.‐P. J. C. R. Park . 2015. “Stem Cell–Derived Bioactive Materials Accelerate Development of Porcine In Vitro–Fertilized Embryos.” Cellular Reprogramming 17, no. 3: 181–190. 10.1089/cell.2014.0110. 26053518 PMC4487679

[vms370179-bib-0034] Lee, Y. L. , K. F. Lee , J. S. Xu , et al. 2004. “The Embryotrophic Activity of Oviductal Cell‐Derived Complement C3b and iC3b, a Novel Function of Complement Protein in Reproduction.” Journal of Biological Chemistry 279, no. 13: 12763–12768. 10.1074/jbc.M311160200. 14699127

[vms370179-bib-0035] Li, M. , D. Liu , L. Wang , W. Wang, A. Wang, and Y. Yao. 2016. “Expression of Placenta‐Specific 8 in Human Oocytes, Embryos, and Models of In Vitro Implantation.” Fertility and Sterility 106, no. 3: 781–789. 10.1016/j.fertnstert.2016.05.018. 27322877

[vms370179-bib-0036] Li, S. , and W. Winuthayanon . 2017. “Oviduct: Roles in Fertilization and Early Embryo Development.” Journal of Endocrinology 232, no. 1: 1–26. 10.1530/JOE-16-0302. 27875265

[vms370179-bib-0037] Lopera‐Vasquez, R. , M. Hamdi , V. Maillo , et al. 2017. “Effect of Bovine Oviductal Extracellular Vesicles on Embryo Development and Quality In Vitro.” Reproduction (Cambridge, England) 153, no. 4: 461–470. 10.1530/REP-16-0384. 28104825

[vms370179-bib-0038] Machtinger, R. , L. C. Laurent , and A. A. Baccarelli . 2016. “Extracellular Vesicles: Roles in Gamete Maturation, Fertilization and Embryo Implantation.” Human Reproduction Update 22, no. 2: 182–193. 10.1093/humupd/dmv055. 26663221 PMC4755440

[vms370179-bib-0039] Mahdavinezhad, F. , P. Kazemi , P. Fathalizadeh , et al. 2019. “In Vitro versus In Vivo: Development‐, Apoptosis‐, and Implantation‐ Related Gene Expression in Mouse Blastocyst.” Iranian Journal of Biotechnology 17, no. 1: e2157. 10.21859/ijb.2157. 31457046 PMC6697851

[vms370179-bib-0040] Maillo, V. , R. Lopera‐Vasquez , M. Hamdi , A. Gutierrez‐Adan , P. Lonergan , and D. J. T. Rizos . 2016. “Maternal‐Embryo Interaction in the Bovine Oviduct: Evidence From in Vivo and in Vitro Studies.” Theriogenology 86, no. 1: 443–450. 10.1016/j.theriogenology.2016.04.060. 27177963

[vms370179-bib-0041] Ménézo, Y. J. , E. Servy , A. Veiga , A. Hazout , and K. Elder . 2012. “Culture Systems: Embryo Co‐Culture.” Methods in Molecular Biology 912: 231–247. 10.1007/978-1-61779-971-6_14. 22829378

[vms370179-bib-0042] Mohshina, H. , and Y. Gedik . 2021. “Effects of Different Co‐Culture on Bovine In Vitro Embryo Production: An Overview.” Highly Interconnected & Endless Puzzle: Agriculture. *IKSAD Publising home* 139–156.

[vms370179-bib-0043] Nazifi, S. , H. Nazari , H. Hassanpour , E. Ahmadi , and A. Afzali . 2022. “Co‐Culturing or Conditioned Medium of Sertoli Cells: Which One Supports In Vitro Maturation of Bovine Oocytes and Developmental Competency of Resulting Embryos?” Veterinary Medicine and Science 8, no. 6: 2646–2654. 10.1002/vms3.939. 36084303 PMC9677355

[vms370179-bib-0044] Neira, J. A. , D. Tainturier , M. A. Peña , and J. Martal . 2010. “Effect of the Association of IGF‐I, IGF‐II, bFGF, TGF‐Beta1, GM‐CSF, and LIF on the Development of Bovine Embryos Produced In Vitro.” Theriogenology 73, no. 5: 595–604. 10.1016/j.theriogenology.2009.10.015. 20035987

[vms370179-bib-0045] Nikoobin, M. , E. Ahmadi , N. Shams‐Esfandabadi , H. Nazari , and N. Davoodian . 2024. “Bovine Amniotic Membrane Stem Cell‐Conditioned Medium Improves the Culture Conditions for In Vitro Produced Ovine Embryos.” Small Ruminant Research 230: 107172. 10.1016/j.smallrumres.2023.107172.

[vms370179-bib-0046] Okada, H. , Y. Hirose , P. Manonmani , A. Uda , M. Ito , and T. Sankai . 2005. “Characterization of an Immortalized Oviduct Cell Line From the Cynomolgus Monkey (*Macaca fascicularis*).” Journal of Medical Primatology 34, no. 2: 67–72. 10.1111/j.1600-0684.2005.00093.x. 15860112

[vms370179-bib-0047] Park, H. Y. , E. Y. Kim , S. E. Lee , et al. 2013. “Effect of human Adipose Tissue‐Derived Mesenchymal‐Stem‐Cell Bioactive Materials on Porcine Embryo Development.” Molecular Reproduction and Development 80, no. 12: 1035–1047. 10.1002/mrd.22270. 24150974

[vms370179-bib-0048] Perrini, C. , P. Esposti , F. Cremonesi , and A. Lange Consiglio . 2018. “Secretome Derived From Different Cell Lines in Bovine Embryo Production in Vitro.” Reproduction, Fertility and Development 30, no. 4: 658–671. 10.1071/RD17356. 28982475

[vms370179-bib-0049] Raposo, G. , and W. Stoorvogel . 2013. “Extracellular Vesicles: Exosomes, Microvesicles, and Friends.” Journal of Cell Biology 200, no. 4: 373–383. 10.1083/jcb.201211138. 23420871 PMC3575529

[vms370179-bib-0050] Reinhart, K. C. , R. K. Dubey , B. Cometti , P. J. Keller , and M. Rosselli . 2003. “Differential Effects of Natural and Environmental Estrogens on Endothelin Synthesis in Bovine Oviduct Cells.” Biology of Reproduction 68, no. 4: 1430–1436. 10.1095/biolreprod.102.006569. 12606437

[vms370179-bib-0051] Ruijter, J. M. , C. Ramakers , W. M. H. Hoogaars , et al. 2009. “Amplification Efficiency: Linking Baseline and Bias in the Analysis of Quantitative PCR Data.” Nucleic Acids Research 37, no. 6: 45–56. 10.1093/nar/gkp045. PMC2665230 19237396

[vms370179-bib-0052] Sakurai, N. , K. Takahashi , N. Emura , et al. 2016. “The Necessity of OCT‐4 and CDX2 for Early Development and Gene Expression Involved in Differentiation of Inner Cell Mass and Trophectoderm Lineages in Bovine Embryos.” Cellular Reprogramming 18, no. 5: 309–318. 10.1089/cell.2015.0081. 27500421

[vms370179-bib-0053] Salleh, N. 2014. “Diverse Roles of Prostaglandins in Blastocyst Implantation.” Scientific World Journal 2014: 1–12. 10.1155/2014/968141. PMC3925584 24616654

[vms370179-bib-0054] Schmaltz‐Panneau, B. , Y. Locatelli , S. Uzbekova , C. Perreau , and P. Mermillod . 2015. “Bovine Oviduct Epithelial Cells Dedifferentiate Partly in Culture, While Maintaining Their Ability to Improve Early Embryo Development Rate and Quality.” Reproduction in Domestic Animals 50, no. 5: 719–729. 10.1111/rda.12556. 26302033

[vms370179-bib-0055] Seshagiri, P. B. , and B. D. Bavister . 1991. “Glucose and Phosphate Inhibit Respiration and Oxidative Metabolism in Cultured Hamster Eight‐Cell Embryos: Evidence for the “Crabtree Effect”.” Molecular Reproduction and Development 30, no. 2: 105–111. 10.1002/mrd.1080300206. 1954025

[vms370179-bib-0056] Shadmanesh, A. , H. Nazari , A. Shirazi , E. Ahmadi , and N. Shams‐Esfandabadi . 2022. “Human Amniotic Membrane Stem Cells' conditioned Medium Has Better Support for In‐Vitro Production of Bovine Embryos Than FBS.” Reproduction in Domestic Animals = Zuchthygiene 57, no. 2: 173–184. 10.1111/rda.14038. 34741476

[vms370179-bib-0057] Shirazi, A. , A. Bahiraee , E. Ahmadi , H. Nazari , B. Heidari , and S. Borjian . 2009. “The Effect of the Duration of In Vitro Maturation (IVM) on Parthenogenetic Development of Ovine Oocytes.” Avicenna Journal of Medical Biotechnology 1, no. 3: 181–191. 23408235 PMC3558135

[vms370179-bib-0058] Simons, M. , and G. Raposo . 2009. “Exosomes–Vesicular Carriers for Intercellular Communication.” Current Opinion in Cell Biology 21, no. 4: 575–581. 10.1016/j.ceb.2009.03.007. 19442504

[vms370179-bib-0059] Smits, K. , D. I. De Coninck , F. Van Nieuwerburgh , J. Goavere, M. Van Pouke, L. Peelman, D. Deforce, and A. Van Soom. 2016. “The Equine Embryo Influences Immune‐Related Gene Expression in the Oviduct.” Biology of Reproduction 94, no. 2: 36. 10.1095/biolreprod.115.136432. 26740593

[vms370179-bib-0060] Talukder, A. K. , M. B. Rashid , M. S. Yousef , et al. 2018. “Oviduct Epithelium Induces Interferon‐tau in Bovine Day‐4 Embryos, Which Generates an Anti‐Inflammatory Response in Immune Cells.” Scientific Reports 8, no. 1: 7850. 10.1038/s41598-018-26224-8. 29777205 PMC5959944

[vms370179-bib-0061] Tamrin, N. A. M. , R. Zainudin , Y. Esa , et al. 2020. “New Insights on the Evolution of the Sweet Taste Receptor of Primates Adapted to Harsh Environments.” Animals 10, no. 12: 2359. 10.3390/ani10122359. 33321745 PMC7764350

[vms370179-bib-0062] Uhm, S. J. 2023. “Effect of Supplement of SCM in Culture Medium for In Vitro Development of Bovine In Vitro Fertilized Oocytes.” Journal of Animal Reproduction and Biotechnology 38, no. 3: 143–150. 10.12750/JARB.38.3.143.

[vms370179-bib-0063] Wang, H. , Y. Wen , S. Mooney , B. Behr , and M. L. Polan . 2002. “Phospholipase A2 and Cyclooxygenase Gene Expression in Human Preimplantation Embryos.” Journal of Clinical Endocrinology & Metabolism 87, no. 6: 2629–2634. 10.1210/jcem.87.6.8532. 12050227

[vms370179-bib-0064] Wrenzycki, C. , D. Herrmann , L. Keskintepe , et al. 2001. “Effects of Culture System and Protein Supplementation on mRNA Expression in Pre‐Implantation Bovine Embryos.” Human Reproduction 16, no. 5: 893–901. 10.1093/humrep/16.5.893. 11331635

[vms370179-bib-0065] Yan, L. , M. Yang , H. Guo , et al. 2013. “Single‐Cell RNA‐Seq Profiling of human Preimplantation Embryos and Embryonic Stem Cells.” Nature Structural & Molecular Biology 20, no. 9: 1131–1139. 10.1038/nsmb.2660. 23934149

[vms370179-bib-0066] Yaseen, M. A. , C. Wrenzycki , D. Herrmann , J. W. Carnwath , and H. Niemann . 2001. “Changes in the Relative Abundance of mRNA Transcripts for Insulin‐Like Growth Factor (IGF‐I and IGF‐II) Ligands and Their Receptors (IGF‐IR/IGF‐IIR) in Preimplantation Bovine Embryos Derived From Different In Vitro Systems.” Reproduction (Cambridge, England) 122, no. 4: 601–610. 10.1530/rep.0.1220601. 11570968

[vms370179-bib-0067] Yeung, W. , P. Ho , E. Lau , and S. Chan . 1992. “Improved Development of human Embryos in Vitro by a human Oviductal Cell co‐Culture System.” Human Reproduction 7, no. 8: 1144–11499. 10.1093/oxfordjournals.humrep.a137810. 1400940

